# The function of the NADPH oxidase of phagocytes and its relationship to other NOXs in plants, invertebrates, and mammals

**DOI:** 10.1016/j.biocel.2007.10.003

**Published:** 2008

**Authors:** Anthony W. Segal

**Affiliations:** Centre for Molecular Medicine, University College London, 5 University Street, London WC1E 6JJ, UK

**Keywords:** NADPH, Oxidase, Ion, Channel, Blood pressure

## Abstract

The NADPH (nicotinamide adenine dinucleotide phosphate) oxidase (NOX) of ‘professional’ phagocytic cells transfers electrons across the wall of the phagocytic vacuole, forming superoxide in the lumen. It is generally accepted that this system promotes microbial killing through the generation of reactive oxygen species and through the activity of myeloperoxidase. An alternative scenario exists in which the passage of electrons across the membrane alters the pH and generates a charge that drives ions into, and out of, the vacuole. It is proposed that the primary function of the oxidase is to produce these pH changes and ion fluxes, and the issues surrounding these processes are considered. The neutrophil oxidase is the prototype of a whole family of NOXs that exist throughout biology, from plants to man, which might function, at least in part, in a similar fashion. Some examples of how these other NOXs might influence ion fluxes are examined.

## 1 Introduction

The nicotinamide adenine dinucleotide phosphate (NADPH) oxidase (NOX) found in ‘professional’ phagocytes (NOX2) is the prototype of the NOX and dual oxidase (DUOX) family of electron transport chains. Historically, the phagocyte oxidase has been thought to function by generating reactive oxygen species (ROS) that kill microbes and play a role in the pathogenesis of disease by damaging normal tissues. Consequently, when the other NOXs were discovered, they were also thought to have an antimicrobial role through free radical reactions, and in the absence of such a microbicidal function, the ROS were thought to exert their effect through ‘cell signaling’. In this review, I examine the proposal that the phagocyte oxidase and, by analogy, other NOXs have another role. When electrons are transported across membranes, the membranes may be depolarized, and the pH on both sides of the membrane is altered. These changes induce compensatory ion fluxes. Whereas the DUOXs have specialized domains for the dismutation of O_2_^−^, and produce H_2_O_2_ as substrate for peroxidation and other oxidation reactions, I examine the proposal that NOXs may also function to drive these ion fluxes and pH changes, and that the O_2_^−^ is formed as the simplest acceptor of electrons rather than as a specific free radical product.

## 2 The neutrophil NADPH oxidase and the roles and consequences of ion fluxes into the phagocytic vacuole

Phagocytosis by neutrophils is accompanied by a burst of oxygen consumption known as the ‘respiratory burst’. This is non-mitochondrial respiration that is accomplished by the NADPH oxidase, a protein complex that assembles in the wall of the phagocytic vacuole and produces O_2_^−^ in the vacuole (Segal, 2005; Cross & Segal, 2004). Electron transfer occurs through gp91^*phox*^, a flavocytochrome *b* that contains a trans-membrane domain in which there are two low potential hemes, located near the internal and external surfaces of the membrane, and cytosolic domains that bind flavin adenine dinucleotide (FAD) and the substrate NADPH.

Electron transport requires activation by kinases and participation of the cytosolic adapter proteins p47^*phox*^, p67^*phox*^, and Rac2. These cytosolic proteins translocate to the membrane, where they associate with gp91^*phox*^. Modeling of the cytosolic region of gp91^*phox*^ (Taylor, Jones, & Segal, 1993) places a small helix over the NADPH binding site, and activation by these cytosolic factors might involve the displacement of this helix, thereby bringing the substrate into close proximity to the FAD.

Chronic granulomatous disease (CGD) is an immunodeficiency syndrome characterized by a profound susceptibility to bacterial and fungal infection, which is caused by failure of this oxidase system. In approximately two thirds of cases, this disease is caused by a mutation in the gene coding for gp91^*phox*^, in 25% the abnormal protein is p47^*phox*^, and in the remaining small percentage of cases the abnormality is in p67^*phox*^ or in the second subunit of the heterodimeric flavocytochrome *b* called p22^*phox*^ (Thrasher, Segal, & Casimir, 1993).

The activity of the oxidase is completely deficient in the vast majority of cases with CGD. In a small number of cases, mutations in the components of the NADPH oxidase, usually in regulatory regions of the proteins, result in partial loss of the protein, or of its function (Roos et al., 1992). Cells from these patients, with what is known as ‘variant CGD’, can produce up to 10–20% of normal amounts of oxidase activity, and yet these patients present clinically because they are predisposed to infections and other manifestations of CGD. This ‘variant’ condition becomes very relevant when considering the function of the oxidase, as discussed below.

## 3 How does NADPH oxidase activity promote microbial killing?

The NADPH oxidase transfers electrons across the wall of the phagocytic vacuole, where it is accepted by O_2_, forming O_2_^−^ in the vacuole. There is a major difference of opinion as to the role of this electron transport.

### 3.1 The theory of direct toxicity by free radicals and reaction products of myeloperoxidase

It has been known since 1961 that the respiratory burst generates H_2_O_2_ (Iyer, Islam, & Quastel, 1961), and Klebanoff (1967) showed that myeloperoxidase (MPO) was microbicidal when combined with H_2_O_2_ and halides. MPO-mediated halogenation was readily accepted as the primary microbicidal mechanism. Subsequently, the discovery of superoxide dismutase (SOD) (McCord & Fridovich, 1970) ushered in the era of the theory of the toxicity of oxygen radicals in biological systems, because it facilitated the quantitative measurement of O_2_^−^ in a system that was not toxic to cells. Neutrophils were found to produce copious quantities of superoxide (Babior, Kipnes, & Curnutte, 1973), the O_2_^−^ itself, and various reaction products, including H_2_O_2_, OH•, singlet oxygen, and ozone, which were all considered microbicidal, singly or in combination (Klebanoff, 1975, 2005).

The conventional idea is that the O_2_^−^ dismutates to form H_2_O_2_, that these molecules react to form other ROS, such as singlet oxygen and the OH• radical, and that these ROS are directly toxic to the microbe. In addition, MPO, a very abundant protein released from the granules into the vacuole, can catalyze the H_2_O_2_-dependent oxidation of halides to form toxic hypohalous acids.

## 4 Evidence supporting the theory of direct toxicity of ROS and hypohalous acids

### 4.1 Production of O_2_^−^ and H_2_O_2_ in the test tube

Soon after, it was found to be produced by neutrophils, a xanthine/xanthine oxidase system was used to generate O_2_^−^ *in vitro*, which was claimed to kill bacteria directly. Surprisingly, in these experiments, the xanthine oxidase enzyme alone killed the bacteria in the absence of the substrate xanthine (Babior, Curnutte, & Kipnes, 1975). A similar study went on to show that microbial killing in this system was not inhibited by SOD (Klebanoff, 1974) and suggested that the O_2_^−^ was dismutating to produce H_2_O_2_ as substrate for MPO-mediated generation of HOCl.

These studies were performed in the early days after the discovery that O_2_^−^ is produced by neutrophils and before it had been appreciated what enormous amounts of O_2_^−^ and H_2_O_2_ are generated in the vacuole. Although radical production was not accurately quantitated in these experiments, it was likely to be orders of magnitude lower than the amounts generated in the vacuole, or the levels in variant CGD that have proved to be inadequate. Subsequently, it was accepted that O_2_^−^ is ‘surprisingly innocuous and appears to play no role in bacterial killing by neutrophils’ (Gabig & Babior, 1981). H_2_O_2_ was also shown to be fairly ineffectual in killing bacteria, unless present together with ascorbate and free metal ions (Drath & Karnovsky, 1974) which would not be available as free copper and iron are bound in the vacuole by lactoferrin, which is unsaturated in this location (Gutteridge, Paterson, Segal, & Halliwell, 1981; Winterbourn, 1983). Suggestions that neutrophils produce singlet oxygen, which might be toxic, have been seriously questioned (reviewed in Klebanoff, 2005).

A novel mechanism for neutrophil killing was introduced with the concept that immunoglobulin G (IgG) catalyzed the generation of H_2_O_2_ and ozone and that the latter was produced in high concentrations in the phagocytic vacuole (Wentworth et al., 2002). This finding would not explain the failure of bacterial killing in CGD, where IgG-opsonized bacteria are not killed in the vacuole, and in addition, it has been demonstrated recently that the assay for ozone is not specific, as it also measures O_2_^−^ (Kettle, Clark, & Winterbourn, 2004).

### 4.2 Influence of SOD and catalase on bacterial killing

Early killing assays used a simple system in which neutrophils, bacteria, and serum (10%) were mixed in a test tube, which was rotated end over end (Quie, White, Holmes, & Good, 1967). Phagocytosis was determined after 2 h of mixing by measuring the numbers of viable bacteria in the supernatant after centrifuging the cells at 800 × *g* for 5 min. Killing was determined by plating out aliquots of the mixture of cells and bacteria. Phagocytosis was measured by determining the numbers of viable bacteria in the supernatant after centrifugation of the mixture of cells and bacteria, and no account was made of the possibility that some of the organisms might have been killed outside the cells.

Under these conditions, both SOD and catalase were shown to impair bacterial killing very slightly, and this inhibitory effect was accentuated if these enzymes were administered together with latex particles (Johnston et al., 1975). It was suggested that the latex particles were carrying the SOD and catalase into the phagocytic vacuole, where they were more effectively exerting their inhibitory effect. Inhibition by both enzymes was taken to suggest that O_2_^−^ and H_2_O_2_ were reacting to generate OH• in the Haber–Weiss reaction. However, the latex particles impaired phagocytosis and might have been exerting their effect in this way. In addition, these enzymes have not been shown to be active in the phagocytic vacuole, where they are liable to be rapidly digested.

### 4.3 Proposed vulnerability of catalase-negative bacteria to being killed in CGD cells

Catalase-negative organisms rarely infect CGD patients (Gallin et al., 1983), and it was proposed that these bacteria generated enough H_2_O_2_ to catalyze their own MPO-mediated halogenation within the vacuole of the phagocytosing neutrophil (Holmes & Good, 1972; Pitt & Bernheimer, 1974). This theory was supported by a study in which *in vitro* mutagenesis was used to generate strains of *Staphylococcus aureus* containing varying levels of catalase. Their virulence in mice was found to be inversely proportional to their catalase content (Mandell, 1975).

## 5 Evidence against the direct toxicity of ROS and hypohalous acids

### 5.1 The oxidase products are not microbicidal under conditions prevailing in vacuoles

Until recently, no attempt was made to quantitate the ROS generated in the vacuole or to determine the conditions in which they were interacting with the other vacuolar constituents. The amounts of O_2_^−^ produced are enormous, in the order of about 1–4 mol/l (Reeves et al., 2002; Winterbourn, Hampton, Livesey, & Kettle, 2006), the concentration of granule proteins is as much as 500 mg/ml (Reeves et al., 2002), and the pH is between 7.4 and 8.0 (Segal, Geisow, Garcia, Harper, & Miller, 1981). A study was thus undertaken to examine the antibacterial action of O_2_^−^ and H_2_O_2_ and products of chloride oxidation (HOCl) under these conditions (Reeves, Nagl, Godovac-Zimmermann, & Segal, 2003).

It was found that these reactive species did kill bacteria when incubated with them in a protein-free salt solution. However, in the more physiological situation in which granule proteins were also present at a concentration of 25 mg/ml (which was a practical concentration to work with but only amounted to about 5% of that in the vacuole), these proteins provided alternative chemical groups with which the reactive species could react, and this microbicidal effect of the ROS and HOCl was abolished (Reeves et al., 2003).

### 5.2 Catalase mutants

The theory that bacterial mutants that were defective in catalase might cause their own destruction by producing H_2_O_2_ was attractive. However, catalase-deficient *Aspergillus nidulans* (Chang, 1998) and *S. aureus* (Messina, Reeves, Roes, & Segal, 2002) have subsequently been shown to be as virulent as the catalase-positive variety in mouse models of CGD. Experiments were also performed to directly investigate whether the hydrogen peroxide produced by catalase-negative *S. aureus* could be used for MPO-mediated halogenation by CGD neutrophils. It was found that virtually no iodination occurred (Messina et al., 2002), clearly demonstrating that although this theory was attractive, it was found to be incorrect when tested experimentally.

### 5.3 Variant CGD

Initially, all the patients diagnosed with CGD had a complete absence of oxidase activity. Subsequently, unusual individuals presented with clinical symptoms of infection and other manifestations of CGD, and yet their neutrophils were found to exhibit some oxidase activity. The neutrophil oxidase activity can amount to 10% (Roos et al., 1992) and up to 30% in rare individuals (H. Malech, personal communication) of normal activity. The cells demonstrate defective microbial killing, and the patients have symptomatic infections. This finding shows that the production of approximately 100 mM O_2_^−^ in the vacuole is insufficient to kill the microbes, an amount that is considerably greater than would be produced by a xanthine/xanthine oxidase-generating system or by catalase-deficient organisms.

### 5.4 Knockout mice

The strongest evidence against the microbicidal effect of ROS and oxidized halides is provided by mice in which the genes for the neutral granule proteases, cathepsin G, and elastase were removed from the neutrophil granules by gene targeting (Reeves et al., 2002). These mice demonstrated a normal respiratory burst, indicating the normal production of ROS, and normal iodination, as a consequence of normal MPO activity, but showed a marked inability to kill *S. aureus* and *Candida albicans*, with a killing defect at least as severe as that in the CGD mouse lacking p47^*phox*^. Interestingly, whereas *S. aureus* was killed by elastase but not by cathepsin G, the opposite was found to be the case with *C. albicans*.

These results show that the products of the oxidase and the consequences of their reaction with MPO do not to kill the microbes in the vacuole. The neutral proteases are also required but are not themselves sufficient, because they are unable to kill efficiently in the absence of oxidase activity, as in CGD or under anaerobic conditions. Thus, both the neutral proteases and adequate activity of the oxidase are required. What is the relationship between these two seemingly diverse systems?

## 6 Role of MPO

**Fig. 1:**
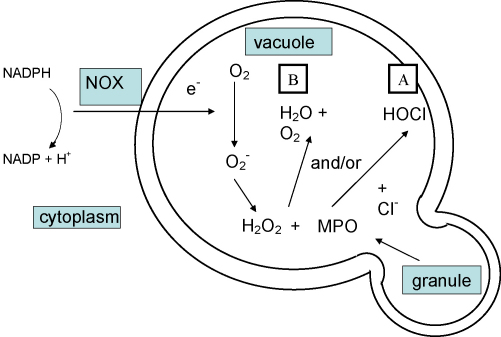
Schematic representation of role of MPO in the vacuole. (A) Currently accepted; (B) Proposed.

If the predominant function of the oxidase is to optimize conditions for the efficient function of the granule enzymes in the phagocytic vacuole, then the current dogma regarding the antimicrobial role of MPO needs to be reconsidered (Fig. 1).

### 6.1 MPO-catalyzed peroxidation reactions in microbicidal processes

There is a vast literature on MPO in neutrophils and its role in microbicidal processes in these cells (reviewed in Klebanoff, 2005), and it is generally accepted that HOCl produced by these cells is the primary killing mechanism. However, a number of recent observations have cast doubt upon the central importance of the products of the oxidase and reaction products of MPO on microbial killing.

As described above, mice targeted for the genes for cathepsin G and elastase do not kill *S. aureus* or *C. albicans* normally, despite normal NADPH oxidase and normal MPO activity, the latter as evidenced by a normal iodination response (Reeves et al., 2002). These experiments indicated that products of the oxidase themselves and the consequences of the action of MPO on these products within the phagocytic vacuole are insufficient to kill the engulfed organism. It is important to stress that these experiments were designed to limit the assessment of killing to that occurring within the phagocytic vacuole, with about 80% of organisms being killed within 4 min of their addition to the cell suspension (Segal et al., 1981).

Although H_2_O_2_ and HOCl can kill *Escherichia coli* and *S. aureus*, this microbicidal effect is dependent upon the conditions under which these compounds are acting. In the past, these killing reactions have been measured in solutions containing little or no protein. The granule proteins achieve a concentration of about 500 mg/ml in the vacuole (Reeves et al., 2002). The toxicity of 1 mM HOCl for *E. coli* and *S. aureus* was completely abrogated by the addition of 25 mg/ml of such proteins (Reeves et al., 2002). H_2_O_2_ (100 mM) killed *S. aureus* in 150 mM NaCl, but this microbicidal effect was abolished, rather than accelerated, when 5 mg/ml MPO was added, strongly suggesting that the MPO was acting as a catalase rather than by generating HOCl (Reeves et al., 2003). This catalase activity of MPO has been measured (Kettle & Winterbourn, 2001). It is promoted when superoxide is present to reduce compound II back to the native enzyme, as would occur in the vacuole.

MPO deficiency occurs in approximately one in 2000–4000 people in the general population (Parry, Root, Metcalf, Delaney, & Kaplow, 1981), and there is no evidence that it is associated with an increased incidence of infection. Genetically targeted MPO-deficient mice kill *S. aureus* normally, although they do demonstrate an increased susceptibility to *Candida* infection (Aratani et al., 1999). This susceptibility could be related to a differential effect of MPO within and outside the vacuole (as discussed below). Lymphopenic mice developed spontaneous infections, if deficient in NADPH oxidase but not if they lacked MPO (Ostanin, Barlow, Shukla, & Grisham, 2007).

The amount of superoxide pumped into the vacuole is enormous, estimated at between 1 and 4 mol/l (Hampton, Kettle, & Winterbourn, 1998; Reeves et al., 2002), producing about 0.5–2.0 mol/l H_2_O_2._ If the estimates that between 28% (Foote, Goyne, & Lehrer, 1983) and 72% (Thomas, Grisham, & Jefferson, 1983) of the H_2_O_2_ is converted into HOCl are correct, this would generate 100 s of mmol/l of HOCl. It is difficult to imagine that this production would be anything but enormously damaging to the vacuolar enzymes, the cell, and the surrounding tissues. HOCl has been shown to inactivate neutrophil neutral proteases and microbicidal enzymes at concentrations of 100–800 μM (Vissers & Winterbourn, 1988). It has been modeled to be produced in the vacuole at 134 mM/min, a concentration predicted to destroy all the granule enzymes (Winterbourn et al., 2006). It appears biologically uneconomical to produce a complex array of microbicidal and digestive proteins, only for them to be degraded as soon as they enter the vacuole. The argument has been produced that degradation of these proteins plays an important role in reducing the potential for these enzymes to damage the surrounding normal cells (Clark & Borregaard, 1985; Hirche, Gaut, Heinecke, & Belaaouaj, 2005), but this rationale fails to take into account what happens in the vacuole.

In addition, a clear anomaly exists between our experiments using flurosceinated bacteria and those of some others. We were the first to use fluroscein-coated, IgG-opsonized *S. aureus* to determine the pH within the phagocytic vacuole of neutrophils (Segal et al., 1981) and did not see any evidence of HOCl-induced bleaching reported subsequently by others (Jankowski, Scott, & Grinstein, 2002). In pilot experiments, we have found that 10 mM HOCl bleaches these organisms, so a major discrepancy exists between the theoretical production of HOCl and our observations of its likely vacuolar concentration.

Patients with variant CGD (Roos et al., 1992) are identified because they are clinically symptomatic with infections, indicating that they are unable to kill bacteria normally *in vivo*, and a microbicidal defect is also observed *in vitro*. A conservative estimate of H_2_O_2_ normally produced in the vacuole is about 500 mM (from the dismutation of 1 M O_2_^−^), which means that these patients are producing about 50 mM H_2_O_2_. If only 20% of this H_2_O_2_ is converted into HOCl in these patients, this amounts to about 10 mM, about two orders of magnitude greater than that necessary to kill bacteria in the test tube.

An important part of the theory of MPO-mediated toxicity is that HOCl damages normal cells adjacent to the acute inflammatory process involving the neutrophils. Generation of the atheromatous plaque is associated with inflammation in the vessel wall, and it was predicted that the development of atheroma would be diminished in mice targeted for the MPO gene. In fact, quite the opposite was observed. When the study was performed by repopulating the bone marrow of lethally irradiated low density lipid receptor-deficient mice with bone marrow from MPO-deficient mice, the atheromatous lesions were found to be about 50% larger than in the wild-type mice (Brennan et al., 2001). This was interpreted as indicating an unexpected protective role for MPO-generated reactive intermediates in murine atherosclerosis. An alternative explanation is that MPO protects enzymes, which normally degrade the lipids that accumulate in atheromatous plaques, from oxidative damage by neutrophil- or monocyte/macrophage-derived H_2_O_2_.

For HOCl to exert its antimicrobial effect, it must react with the microbe. Differing results have been obtained in experiments directed to demonstrate this interaction. Whereas evidence has been produced to demonstrate chlorination of ingested organisms (Jiang, Griffin, Barofsky, & Hurst, 1997; Painter et al., 2006), studies to identify the targets of halogenation reactions in phagocytosed bacteria found that it was the proteins of the neutrophil granules, rather than the ingested organisms, that were iodinated (Segal, Garcia, Harper, & Banga, 1983; Reeves et al., 2003) or chlorinated (Chapman, Hampton, Senthilmohan, Winterbourn, & Kettle, 2002).

The conditions within the vacuole do not favor MPO-mediated peroxidase reactions. The pH optimum of these reactions is at about pH 5.0 (Klebanoff, 1967), whereas the pH in the vacuole at the time microbial killing occurs is 7.8–8.0 (Segal et al., 1981), at which pH microbial killing *in vitro* by the MPO, H_2_O_2_ halide system *in vitro* was not seen (Klebanoff, 1967).

The NADPH oxidase of neutrophils, NOX2, is a member of the rapidly growing family of NOXs (Lambeth, 2002). These flavocytochromes conform to the basic structure that has been elucidated for NOX2, with a cytosolic flavoprotein linking to a pair of hemes that span and transfer electrons across the plasma membrane. Some of these molecules have been called DUOXs, because they have the standard NOX domain coupled to a domain with homology to that of thyroid peroxidase. In fact, this ‘peroxidase’ domain has no peroxidatic activity but functions as a SOD to produce H_2_O_2_ from the O_2_^−^ product of electron transport. Is it possible that myeloperoxidase serves a similar function in the NOX2 system?

Besides its HOCl-generating capacity, MPO can act as a catalase, a peroxidase, and a generator or consumer of superoxide, peroxide, and oxygen. The balance of these reactions will be very dependent upon ambient conditions of pH, ionic strength, and enzyme and substrate concentrations, and these conditions have been modeled recently (Winterbourn et al., 2006). MPO is present in a number of different environments in which the reactions it undertakes might differ considerably.

### 6.2 The phagocytic vacuole

MPO is present at very high concentrations within the vacuole. It comprises about 20% of the total granule protein and is released into the vacuole at about 100 mg/ml, where it is exposed to high concentrations of H_2_O_2_ and also of O_2_^−^. These conditions could result in catalase and dismutase, rather than peroxidase, types of reactions via the reaction pathways summarized above and as modeled by Winterbourn and co-workers (Kettle & Winterbourn, 2001; Winterbourn et al., 2006). In addition, little is known of the temporal relationship in the vacuole between the products of the oxidase, degranulation of the granule contents, and the subsequent interaction between the two. In particular, we know that the pH of the vacuole is elevated initially and then slowly declines, becoming acidic after about 15 min. It is not known whether there is a slow rate of oxidase activity at this stage when a peroxidase type of reaction is more likely.

### 6.3 At locations of frustrated phagocytosis

The neutrophil is too small to engulf some of the microbes that it is required to kill, such as fungal mycelia. Under these circumstances, it attaches to the surface of the organism onto which it discharges its granules and the products of the oxidase. The attachment of the neutrophil can be so tight as to provide a virtually closed compartment where the conditions differ little from those of the vacuole. Under other circumstances, the activated membrane can be relatively opened to the extracellular medium. In this case, the concentrations of MPO and H_2_O_2_ will be much lower, and O_2_^−^ would be less available for regeneration of MPO, encouraging peroxidase rather than catalase types of reactions. This case could explain why the MPO knockout mouse is susceptible to fungal but not bacterial infections (Aratani et al., 2000).

### 6.4 In the test tube

Most of the experiments on the activity of MPO are performed on cells in a test tube, leading to variable conditions that make the results heterogeneous and difficult to interpret. In essence, a combination of the two environments described above can occur. Where the particles are greatly in excess or poorly opsonized or where slow shaking or stirring results in a low collision frequency, low levels of MPO and H_2_O_2_ can be released into the extracellular medium (Quie et al., 1967). Under these circumstances, peroxidation of the extracellular particles will occur. Some of these particles will subsequently be phagocytosed, making it difficult to differentiate between reactions that take place inside or outside the vacuole.

## 7 The oxidase elevates the pH in and induces ion fluxes across the membrane of the phagocytic vacuole

**Fig. 2:**
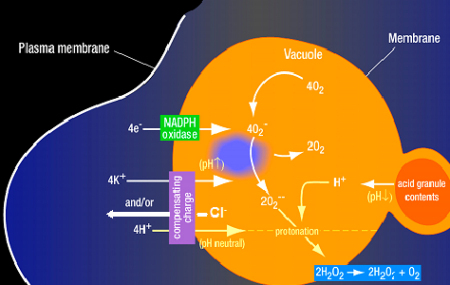
NADPH oxidase induces a charge across the vacuolar membrane that must be compensated. The compensating ions influence the vacuolar pH.

**Fig. 3:**
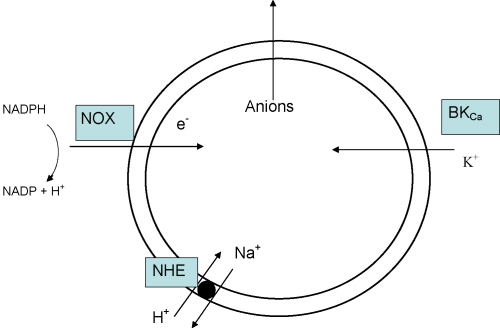
Schematic representation of the ion fluxes induced by the NADPH oxidase in neutrophils, which might act as a generic scheme for the NOXs in general.

An alternative concept is that the main function of electron transport is to drive ion fluxes across the vacuolar membrane and to adjust the pH within the vacuole, so as to optimize conditions for the killing and digestive functions of the granule enzymes (Figs. 2 and 3). The oxidase is electrogenic (Henderson, Chappell, & Jones, 1987), which means that electron transport across the wall of the vacuole generates a charge across the membrane that rapidly becomes of sufficient magnitude to stop further electron flow, unless the charge is compensated by the passage of positively charged ions into the vacuole from the cytoplasm, or negatively charged ions in the opposite direction.

The electrons are released from NADPH, and for each electron that passes across the membrane, one H^+^ is left in the cytoplasm, causing a fall in cytosolic pH, particularly adjacent to the respiring membrane. It has been believed for many years that that the charge across the vacuolar membrane is compensated completely by the passage of these protons into the vacuole from the cytosol, through specific proton channels (DeCoursey, Morgan, & Cherny, 2003) or through the flavocytochrome, gp91^*phox*^ itself (Henderson & Meech, 2002; Banfi et al., 1999). In addition to compensating the charge across the membrane, this process would achieve the effects of removing the H^+^ ions left behind in the cytosol.

## 8 The proton channel

Theories relating to charge compensation of the respiratory burst by H^+^ flux through this channel has recently been reviewed (DeCoursey, 2003; Murphy & DeCoursey, 2006). There is no doubt that neutrophils do contain a proton channel that is constitutively open, the point of debate is whether or not it is the exclusive charge-compensating channel. There is no firm evidence linking it to any role in charge compensation of the oxidase. This channel is very sensitive to inhibition by Zn^2+^, which is active in the low μM range in electrophysiological studies. However, Zn^2+^, in the mM range, does not inhibit the oxidase if added to intact neutrophils at the same time as the activating stimulus (Ahluwalia et al., 2004). Previous suggestions that Zn^2+^ caused inhibition of the oxidase (DeCoursey et al., 2003) were shown to be an artefact of the assay (Ahluwalia et al., 2004). When analyzing the effects of this element on neutrophils, care must be taken not to incubate it with the cells for any length of time, as it is toxic, which makes differentiating between a specific effect and toxicity very difficult.

A voltage-gated H^+^ channel, H(v)1, has been identified that is thought to represent the proton channel that compensates the charge in neutrophils (Ramsey, Moran, Chong, & Clapham, 2006; Sasaki, Takagi, & Okamura, 2006). Interestingly, the expression pattern was unlike one that might be expected from a protein strongly associated with a highly specialized process in professional phagocytes. On Northern blots, the main site of RNA production was in lymph nodes. Westerns blots showed that the protein is also found at high levels in some lymphatic tissues, like the lymph nodes and appendix, but not in the tonsil. Although low levels of protein were present in HL-60 cells induced with dimethyl sulfoxide, it was almost undetectable in peripheral blood leukocytes.

The knockout mouse will provide the definitive answer as to whether or not this proton channel provides the only significant charge-compensating mechanism, because if it does, the respiratory burst should be completely inhibited in its absence (DeCoursey et al., 2003).

## 9 Charge compensation by K^+^ and the controversy surrounding BK channels

Proton channels provide an attractive mechanism of charge compensation, because in addition to compensating the charge across the membrane, this channel would achieve the effects of removing the H^+^ ions left behind in the cytosol when electrons are passed across the membrane. However, the pH within the phagocytic vacuoles rises to about 7.8–8.0 (Segal et al., 1981), despite having approximately 200 mM H^+^ released into them from the granules, in which the pH is maintained at about 5.5 (Styrt & Klempner, 1982). Thus, there must be a net consumption of H^+^s in the vacuole by the protonation of O_2_^−^ (peroxide), which could not occur if each electron were accompanied across the membrane by a proton.

**Figure:**
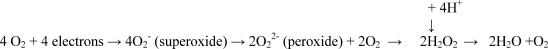

We found that K^+^ ions compensate about 5–10% of the charge (Reeves et al., 2002). This finding was based on the following observations:

(i) when stimulated with phorbol myristate acetate (PMA), an activator of protein kinase C, neutrophils secrete O_2_^−^ extracellularly, and this secretion is accompanied by K^+^ efflux (measured with a K^+^ electrode),
(ii) oxidase-dependent Rb^+^ release (a surrogate for K^+^) was also measured, was consistent with measurements of K^+^ efflux, and was blocked by iberiotoxin (IbTx) and paxilline (see below);
(iii) electron-probe microanalysis showed that the K^+^ concentration in phagocytic vacuoles was much lower cells in which the oxidase was inhibited with diphenylene iodonium (DPI) than it was in the vacuoles of control cells.

The results indicated that the high levels of K^+^ produced in the vacuole as a consequence of oxidase activity activated the neutral proteases and other strongly bound enzymes by displacing them from the negatively charged proteoglycan granule matrix. These solubilized enzymes are then able to kill and digest the organisms. More recently, it has been demonstrated that alkalinization of the vacuole is important in the processing of antigen by dendritic cells, in which the antigen is degraded if the vacuole becomes excessively acidic through failure of NADPH oxidase activity (Jancic et al., 2007; Savina et al., 2006).

## 10 K^+^ ions pass through channels with characteristics similar to those of BK_Ca_ channels

For the reasons described above, we attempted to identify the channel through which the K^+^s were passing into the vacuole.

### 10.1 Inhibitor and opener data

On the basis that compensation of the charge by K^+^ ions rather than H^+^ ions would encourage an elevation in pH, we screened K^+^ channel inhibitors for those that would prevent the oxidase-induced elevation in pH. We found that inhibitors of the BK_Ca_ channel, IbTx and paxilline, caused the pH to fall to the same low levels as those observed when the oxidase was blocked by DPI (Ahluwalia et al., 2004). Another important indicator that the BK_Ca_ channel was involved, was the observation that when NS1619, which opens this channel (Gribkoff et al., 1996), was added, the pH was elevated to about 8.2, well above levels normally reached. The BK_Ca_ inhibitors blocked Rb^+^ release after it had been induced by PMA or NS1619. A number of other K^+^ channel inhibitors (e.g. 4-AP) had no effect (Ahluwalia et al., 2004).

### 10.2 Western blots

Western blot analysis with a polyclonal rabbit antibody to the α-subunit of the human channel (raised against amino-acids 945–961, a segment which largely coincides with the ‘Ca^2+^ bowl’ of BK_Ca_ channel) showed a strong band in eosinophils and neutrophils, mainly in membranes and granules. The apparent molecular mass of this band was lower, about 80 kDa, than that of the standard α-subunit, which is about 120 kDa. However, this channel is very susceptible to proteolysis (Knaus et al., 1995), which could explain these findings.

### 10.3 Polymerase chain reaction, patch–clamp, and Ca^2+^ chelation studies

Polymerase chain reaction (PCR) for the *hSlo* product revealed a band of the appropriate size and correct sequence in HL-60 cells, when these were induced to differentiate into neutrophil-like cells. Patch–clamp studies using the perforated patch technique revealed large currents in neutrophils and in eosinophils in response to PMA, which were inhibited by IbTx and by DPI, but not by Zn^2+^. Cell-attached single-channel recordings showed, as expected, channels with a conductance of about 215 pS. These electrophysiological recordings have been confirmed by others. They made whole cell and single-channel recordings the latter demonstrated an I–V relationship of 210 pS (Yuan, Cai, & Zou, 2006), similar that we obtained (Ahluwalia et al., 2004). Consistent with the involvement of Ca^2+^-dependent K^+^ currents, PMA-induced Rb^+^ efflux was blocked by chelating Ca^2+^ with BAPTA [1,2-bis(o-aminophenoxy)ethane-*N*,*N*,*N*′,*N*′-tetraacetic acid] and EGTA (ethyleneglycotetraacetic acid).

The importance of the BK_Ca_ channel activity was demonstrated by the effect of IbTx and paxilline on neutrophil function. They both completely inhibited killing of *S. aureus*, *S. marcescens*, and *C. albicans* and the digestion of radiolabeled *S. aureus*.

Two studies have recently been published in which the authors have been unable to reproduce our results on the BK channel, both the electrophysiology and the microbial killing (Femling et al., 2006; Essin et al., 2007).

The BK, or BK-like, channel in neutrophils is not constitutively open, and an active oxidase is required before it can be demonstrated. In the study by Essin et al., the same solutions were used as in our study, but we do not know when the stimulus of the oxidase, PMA, was added to the cells. This could be crucial because the oxidase only runs for a finite time. DeCoursey used completely different solutions, which included TMAMeSO_3_, which they have used to block all channels, including K^+^ channels, other than proton channels (Morgan et al., 2003).

### 10.4 Bacterial killing studies

Femling et al. (2006) and Essin et al. (2007) were both unable to repeat our observation that IbTx inhibits the killing of *S. aureus* and *C. albicans* by neutrophils. The methods they employed differed widely from those we described. This is important because both IbTx a polypeptide, and paxilline, an indole-diterpene, are biological molecules and their stability has not been established under the conditions they used, where incubations were prolonged and in the presence of serum, whereas ours were very brief and used IgG opsonised microbes.

## 11 The NOX family of NADPH oxidases

A large family of NADPH oxidases have been identified in fungi, plants, flies, worms, sea urchins, and in multiple organs in higher animals (Bedard & Krause, 2007) by screening databases for proteins homologous to gp91^*phox*^. The central electron-transporting molecules all share a similar structure to that we described for gp91^*phox*^. They have NADPH and FAD-binding sites in the cytosolic C-terminal tail and six transmembrane helices with ligands for the two hemes, one near each surface of the membrane. Some members of the family, called DUOXs (Donko, Peterfi, Sum, Leto, & Geiszt, 2005), have additional domains towards their N-termini that have been called ‘peroxidase’ domains because of their homology with peroxidases such as MPO (Edens et al., 2001). In fact, these domains catalyse the dismutation of O_2_^−^ to H_2_O_2_, so they should be called ‘dismutase’ rather than ‘peroxidase’ domains. They also contain EF-hand calcium-binding domains in a cytoplasmic loop.

### 11.1 DUOXs produce H_2_O_2_ as substrate for peroxidase reactions

Because of the concept that the neutrophil oxidase generates O_2_^−^ to kill microbes, it was assumed initially that the other NOXs had a similar function, and they were ascribed an antimicrobial role. When such a function proved unconvincing, other rationales for free radical generation were proposed, principal among which have been cell signaling functions.

Much more clearly defined roles have presented themselves for the DUOXs, which generate H_2_O_2_ as a substrate for peroxidase-mediated reactions. This H_2_O_2_ is required in worms (Edens et al., 2001) and flies (D. Lambeth, personal communication) for the cross-linking of tyrosines in the cuticle and wing membrane, respectively.

The generation of H_2_O_2_ by the sea urchin egg following fertilization, which is catalyzed by the DUOX Udx1 (Wong, Creton, & Wessel, 2004), has been studied in some detail. The H_2_O_2_ acts as substrate for ovoperoxidase, which cross-links the envelope into a hardened matrix that is insensitive to biochemical and mechanical challenges, providing a permanent physical obstruction to polyspermy (LaFleur, Horiuchi, & Wessel, 1998). How is the charge compensated for upon this transport of electrons to the exterior upon fertilization? There is a great increase in permeability to chloride (Christen, Sardet, & Lallier, 1979) and activation of a Na^+^/H^+^ exchanger, resulting in alkalinization of the cytoplasm and an influx of Na^+^ (Shen & Steinhardt, 1979).

Duox2 and possibly also Duox1 are expressed in the thyroid gland, where they generate H_2_O_2_ as substrate for thyroperoxidase-mediated iodinaton of tyrosyl residues within thyroglobulin (Lacroix et al., 2001). The iodide is transported into the thyroid cell by NIS, the sodium iodide symporter, which co-transports two sodium ions (Na^+^) along with one iodide ion (I^−^), with the transmembrane sodium gradient serving as the driving force for iodide uptake. The mechanism responsible for iodide efflux across the apical membrane into the lumen of the follicle is unknown, but it involves pendrin, a protein that is abnormal in Pendred's syndrome, characterized by goiter and sometimes associated with hypothyroidism and sensorineural deafness. Pendrin (SLC26A4) is a member of the SLC26 family of anion exchangers (Mount & Romero, 2004), and its loss results in abnormalities in the cochlea in which the endolymph is abnormally acidic and contains high levels of Ca^2+^ (Wangemann et al., 2007).

### 11.2 NOXs as pH modulators and drivers of ion fluxes

NOXs are widely distributed throughout the biological world in which they are undoubtedly playing many different roles. I examine a few examples to extend the thesis that an important, if not primary, role of these molecules is to couple electron transport to pH modulation and ion fluxes, as has been demonstrated in the paradigm of the neutrophil vacuole.

### 11.3 NOX3 in the inner ear

NOX3 is strongly expressed in the inner ear. The phenotype of the mouse in which this system is defective has been very revealing (Paffenholz et al., 2004). The mutation in mice from three different mutagenized lines showing the same abnormally ‘tilted’ position of the head and abnormal performance in several motor coordination tests were located in the gene coding for NOX3. The reason for their distorted perception of position was the fact that they failed to calcify their otoconia.

Otoconia are free floating bodies that lie above the sensory hair cells of the utricle and saccule, and movement of the head is normally perceived by pressure of the relatively inert mass on the hair cells. They are formed by the calcification of OC-90/95, a 90–95 kDa glycoprotein (Hughes, Thalmann, Thalmann, & Ornitz, 2006) with homology to secretory phospholipase A2 (Pote et al., 1993), by the deposition of calcium carbonate. The otoconial protein matrix appears to be present in these NOX3 mutant mice, which prompts the question as to how a lesion in the flavocytochrome might cause a failure of otoconial calcification? Because we know that NOX2 in neutrophils causes the vacuole to be alkalinized and to charge compensation by K^+^, a strong possibility must be that NOX3 behaves in a similar fashion to alkalinize the endolymph and possibly also pump Ca^2+^ into the inner ear. Calcium is much less soluble under alkaline conditions, so the failure to alkalinize the endolymph, possibly coupled to a failure to increase the Ca^2+^ concentration, might be responsible.

In the pendrin knockout mouse, the endolymph is excessively acidic, and the Ca^2+^ concentration is abnormally high (Wangemann et al., 2007). There is an almost complete absence of normal otoconia, with frequent giant otoconia, indicative of abnormal endolymph (Everett et al., 2001). In addition, the inner ears are dilated, suggesting an osmotic effect of Cl^−^ trapped in the absence of this anion exchanger.

The co-existence of pendrin with DUOX in the thyroid and with NOX3 in the inner ear, with clear linkage of dysfunction of these two proteins to grossly similar phenotypes, suggests that they might be closely linked mechanistically. It seems very possible that pendrin could be involved in ion transport coupled to charge compensation of electron transport through the DUOX or NOX.

### 11.4 Root hairs

Plant roots produce small hairs, which are long, thin tubular outgrowths from epidermal cells that are produced in the differentiating zone of the root (Carol & Dolan, 2002). A lot of work has been done on the mechanisms underlying root hair growth. Extension occurs at the tip where the cell wall is thin and bulges, as if under internal pressure. It is relatively plastic in this region, hardening behind the tip. The hair also contains a large vacuole, which might play a role in the extension process. It responds rapidly to changes in extracellular osmotic potential, with hyperosmotic treatment inducing very large increases in ionic conductance across the wall of the vacuole.

Inward ion fluxes are required to maintain the tugor pressure that is believed to be the driving force in cellular expansion (Cosgrove, 1986). A number of different ion fluxes occur across the plasma membrane. The main driving force is an H^+^-ATPase that generates an electrochemical proton gradient across the plasma membrane (Sondergaard, Schulz, & Palmgren, 2004), which induces K^+^ uptake through TEA-sensitive channels (Lew, 1991).

It is also possible that a transmembrane electron transport system is responsible for membrane depolarization (Grabov & Bottger, 1994) driving K^+^, Cl^−^ (Lew, 1991), and other ion fluxes that are necessary for increasing the osmotic pressure in the region of the tip of the root hair, thereby driving it forward.

*Arabidopsis* contains various respiratory burst oxidase homologues, which also contain EF-hand Ca^2+^-binding motifs in their extended N-terminal regions (Torres et al., 1998). One such homologue, RHD2, is important for root development. When the gene coding for it is knocked out (Foreman et al., 2003), the roots are stunted, and the root hairs that develop are very short. RHD2 produces ROS at the root hair tip, and it was suggested that the ROS induce root hair extension by opening hyperpolarization-activated Ca^2+^ channels. The mechanism by which Ca^2+^ gradients might induce root hair extension has not been established.

### 11.5 NOX enzymes in mammals

In addition to NOX2 in phagocytes and NOX3 in the inner ear, described above, mammals contain NOXs 1, 3, and 5. NOX1 is expressed most highly in the colon and NOX4 in the kidney and blood vessels. These are sites in which there is an interface between cells and a surface across which major fluxes of ions occur, in which the NOX system might be mechanistically involved.

### 11.6 Vasculature

NOXs are expressed in endothelial, adventitial, and smooth muscle cells of the vasculature (reviewed in Bedard & Krause, 2007), and there is a large literature of the role of NOXs and ROS in the regulation of blood pressure. The general opinion is that NOXs do have an important regulatory role on the vasculature, including an effect on vascular tone and blood pressure regulation.

The NOXs are thought to exert their effects in a number of ways. Signaling is thought to be regulated directly by the oxidation or reduction of signaling proteins, like protein tyrosine kinases and phosphatases and mitogen-activated protein kinases, and by activating transcription factors, such as nuclear factor-κB and activator protein-1. In addition, ROS produced by the NOXs are thought to react with NO to negate its vasodilatory activity.

NOX1 appears to be involved in the hypertensive response to angiotensin II (Matsuno et al., 2005).

It is generally believed that blood pressure is regulated by contraction of the muscular wall of the resistance vessels. Another possibility is that blood pressure is regulated, at least in part, by the osmotic swelling and shrinkage of cells in response to fluxes of ions driven by NOX-induced charge compensation and associated NHE activity. According to Hagen–Poiseuille's law, the resistance to fluid flow through a vessel is related to the fourth power of the radius, as a consequence of which minor alterations in the size of the cells, either in the smooth muscle or endothelium, can cause significant alterations in resistance and consequently in blood pressure.

This process could be driven and regulated by NOXs and could occur in any of the cells in the vessel walls in which these electron transport chains are expressed. On the basis of the model system described above, the efflux of electrons would be balanced by the influx of Cl^−^ and the exchange of intracellular H^+^s for extracellular Na^+^ through sodium proton exchangers, followed by the osmotic movement of water. The inhibition of the influx of Na^+^ through NHEs, which are inhibited by thiazide diuretics, could explain the antihypertensive effect of these drugs, which appear to exert their action through vasodilatation rather than saluresis or loss of free water (Conway & Lauwers, 1960; van, Man in ’t Veld, & Schalekamp, 1980).

## 12 Summary

In the neutrophil, electron transport through the NADPH oxidase of phagocytic cells depolarizes the membrane of the phagocytic vacuole and acidifies the cytosol, whereas the generation of O_2_^−^ in the vacuole alkalinizes this compartment. These changes induce secondary ion fluxes, which result in activation of the granule proteins discharged into the vacuole and restitution of the excessive ion and pH imbalances. It had been assumed, by analogy with the phagocyte oxidase, that other NOXs were serving an antimicrobial function or that the free radicals were generated in a signaling role. The demonstration that NOX2 is working by inducing ion fluxes serves as an example of the possible ways in which NOXs in other situations in biology might be functioning.
